# Editorial: Neurotrophins and their importance on neural plasticity: New insights and potential therapeutic effects on brain pathology

**DOI:** 10.3389/fnmol.2022.1082116

**Published:** 2022-11-15

**Authors:** Marta Perez-Rando, Esther Castillo-Gomez

**Affiliations:** ^1^Neurobiology Unit, Program in Neurosciences and Institute of Biotechnology and Biomedicine (BIOTECMED), Universitat de Valéncia, Valencia, Spain; ^2^Spanish National Network for Research in Mental Health, Centro de Investigación Biomédica en Red de Salud Mental (CIBERSAM), Madrid, Spain; ^3^Fundación Investigación Hospital Clínico de Valencia, INCLIVA, Valencia, Spain; ^4^Department of Medicine, School of Medical Sciences, Universitat Jaume I, Castellón de la Plana, Spain

**Keywords:** neurotrophic factors, TrkB, Val66Met, nerve growth factor (NGF), brain-derived neurotrophic factor (BDNF), brain plasticity

Neurotrophins, or neurotrophic factors, are molecules that promote the survival and development of neurons. Their discovery started with the breakthrough description of the nerve growth factor (NGF) by Rita Levi-Montalcini and Stanley Cohen back in 1951, a molecule that promoted the survival and elongation of nerves in chicken (Levi-Montalcini and Hamburger, 1951). This was followed by many important findings in the field, and finally led to their Nobel Prize back in 1986. These years of intense research led in 1982 to the discovery of the brain-derived neurotrophic factor in mammals (BDNF), which revolutionized the field (Barde et al., 1982). With the detailed description of many neurotrophic factors came the discovery of their receptors. Among this, we need to highlight tyrosine protein kinase B (TrkB), the BDNF receptor (Klein et al., 1989).

Other approaches have focused on genetic expression and translation of neurotrophic factors. One example of the great advance on this topic is the discovery of the Val66Met mutation on the BDNF gene. This mutation disrupts BDNF release leading to a variety of cellular and subcellular alterations and has been proposed to affect the vulnerability to undergo psychiatric disorders. However, more research is needed to fully understand its role. In this Research Topic, Williams et al. study how isoflurane causes a transient and reversible inhibition of exocytosis of excitatory vesicles, fact that seems to be mediated by BDNF. However, the presence of the Val66Met mutation on the BDNF gene prevents this recovery from happening.

Thanks to this knowledge and with the continuous information influx, novel drugs have been presented that could work finely promoting the neurotrophin effect, providing researchers with the tools to dig deeper into the biology of these proteins. This is the scientific approach that Tacke et al. use in their study. Here they used the TrkB agonist ZEB85 to investigate its role on activity and plasticity of culture neurons. Importantly, this molecule also prevents β-amyloid toxicity in cultured hippocampal neurons, a piece of information that adds to our fight against Alzheimer's disease (Tacke et al.).

On the other hand, although their presence and influence are more prominent during development, neurotrophins also trigger plasticity phenomena in the adult brain and are highly disturbed in many neuropsychiatric disorders (Castrén, 2014). In fact, many animal models of neurological and psychiatric disorders detect disturbances in different neurotrophic factors and can rescue some phenotypic deficits administering them. In this regard, Qi et al. prepared an autism mouse model using valproic acid administration and reported its effect on BDNF expression. Furthermore, they rescued the reported synaptic deficits *via* BDNF administration using minipumps during postnatal development (Qi et al.). Last of all, Colitti et al. prepared a stroke model using a brain lesion, which caused a decrease in neurogenesis in the rat SVZ. Importantly, in this article they used intranasal NGF administration to promote neuron growth, and found it triggered neuron maturation (Colitti et al.).

Altogether these articles set the basis for understanding the role neurotrophic factors have on neuron growth and survival, and how their administration can palliate many deleterious phenotypes present if different neural and psychiatric disorders.

## Author contributions

All authors listed have made a substantial, direct, and intellectual contribution to the work and approved it for publication.

## Conflict of interest

The authors declare that the research was conducted in the absence of any commercial or financial relationships that could be construed as a potential conflict of interest.

## Publisher's note

All claims expressed in this article are solely those of the authors and do not necessarily represent those of their affiliated organizations, or those of the publisher, the editors and the reviewers. Any product that may be evaluated in this article, or claim that may be made by its manufacturer, is not guaranteed or endorsed by the publisher.
